# Early Diagnosis of Pneumonia in Severe Stroke: Clinical Features and the Diagnostic Role of C-Reactive Protein

**DOI:** 10.1371/journal.pone.0150269

**Published:** 2016-03-03

**Authors:** Anushka Warusevitane, Dumin Karunatilake, Julius Sim, Craig Smith, Christine Roffe

**Affiliations:** 1 Stoke Stroke Research Group, University Hospitals of North Midlands, Stoke-on-Trent, United Kingdom; 2 Stroke Unit, Taunton and Somerset NHS Trust, Taunton, United Kingdom; 3 Health Services Research Unit, Keele University, Stoke-on-Trent, United Kingdom; 4 Stroke and Vascular Research Centre, University of Manchester, Salford Royal NHS Foundation Trust, Manchester Academic Health Science Centre, Manchester, United Kingdom; Ottawa Hospital Research Institute, CANADA

## Abstract

**Background:**

Accurate diagnosis of pneumonia complicating severe stroke is challenging due to difficulties in physical examination, altered immune responses and delayed manifestations of radiological changes. The aims of this study were to describe early clinical features and to examine C-reactive protein (CRP) as a diagnostic marker of post-stroke pneumonia.

**Methods:**

Patients who required nasogastric feeding and had no evidence of pneumonia within 7 days of stroke onset were included in the study and followed-up for 21 days with a daily clinical examination. Pneumonia was diagnosed using modified British Thoracic Society criteria.

**Results:**

60 patients were recruited (mean age 77 years, mean National Institutes of Health Stroke Scale Score 19.47). Forty-four episodes of pneumonia were identified. Common manifestations on the day of the diagnosis were new onset crackles (43/44, 98%), tachypnoea>25/min (42/44, 95%), and oxygen saturation <90% (41/44, 93%). Cough, purulent sputum, and pyrexia >38°C were observed in 27 (61%), 25 (57%) and 15 (34%) episodes respectively. Leucocytosis (WBC>11,000/ml) and raised CRP (>10 mg/l) were observed in 38 (86%) and 43 (97%) cases of pneumonia respectively. The area under the ROC curve for CRP was 0.827 (95% CI 0.720, 0.933). The diagnostic cut-off for CRP with an acceptable sensitivity (>0.8) was 25.60 mg/L (Youden index (*J*) 0.515; sensitivity 0.848; specificity 0.667). A cut-off of 64.65 mg/L had the highest diagnostic accuracy (*J* 0.562; sensitivity 0.636; specificity 0.926).

**Conclusion:**

Patients with severe stroke frequently do not manifest key diagnostic features of pneumonia such as pyrexia, cough and purulent sputum early in their illness. The most common signs in this group are new-onset crackles, tachypnoea and hypoxia. Our results suggest that a CRP >25 mg/L should prompt investigations for pneumonia while values >65 mg/L have the highest diagnostic accuracy to justify consideration of this threshold as a diagnostic marker of post-stroke pneumonia.

## Introduction

Diagnosis of pneumonia is usually based on clinical, biochemical and microbiological variables, and, in some guidelines, a chest radiograph (CXR) [1,2]. However, in stroke patients, especially in those with severe neurological deficits, diagnosis can be difficult. There is considerable variation in diagnostic approach and uncertainty about the cut-off levels for diagnostic variables [3,4]. The risk of infection is increased due to advanced age, dysphagia, co-morbidities, and stroke-induced immunosuppression [5,6]. An altered level of consciousness, receptive dysphasia, respiratory muscle paralysis, and an impaired cough affect the compliance required for physical examination of the respiratory system in stroke patients [3,7]. Changes in acute-phase reactants are seen early after stroke, even in the absence of infection, and are driven largely by the extent of the tissue damage [8]. The CXR may be of limited value in the early stages, as infiltrates take time to develop [9]. Poor positioning and reduced voluntary deep inspiration due to drowsiness can further interfere with the quality and interpretation of the CXR [9]. Therefore, in clinical practice diagnostic and therapeutic decisions relating to pneumonia are mainly based on clinical information [10]. Current treatment guidelines promote early diagnosis and treatment to prevent complications and associated mortality [11]. However, no study has assessed the symptomatology of post-stroke pneumonia for early diagnosis. C-reactive protein (CRP) is an acute-phase protein, which increases with bacterial infections and could therefore be of diagnostic value [6]. However, increases of CRP are also seen in non-infective pathologies such as pulmonary embolism, trauma and malignancies [6]. The aims of this study were 1) to identify clinical signs and symptoms for early diagnosis of post-stroke pneumonia, 2) to assess the usefulness of CRP as a marker of post-stroke pneumonia and 3) to identify an optimal diagnostic cut-off for CRP.

## Methods

This study is a secondary analysis of data from the MAPS (metoclopramide to prevent pneumonia in stroke patients fed via nasogastric tubes) trial [12]. The MAPS trial was conducted between September 2008 and September 2011 on the acute stroke unit of the University Hospital of North Midlands, United Kingdom. The protocol was approved by the North Staffordshire Research Ethics Committee (Approval Number: 07/Q2604/41). Written informed consent or assent was obtained from patients or their next of kin to review patients’ medical notes and to conduct laboratory investigations and chest radiographs for the diagnosis of pneumonia. In addition, all data were anonymized prior to analysis. Patients with acute ischaemic or haemorrhagic stroke who required nasogastric feeds and had no evidence of infection were recruited within 7 days of symptom onset. Baseline observations, inflammatory markers (when available) and chest signs were recorded in all patients on admission, on day 7, day 14, and day 21. Prospective follow-up to screen for symptoms and signs of pneumonia was for 21 days with a daily clinical review and chest examination conducted by the same team. If symptoms or signs suggestive of pneumonia were identified, they were recorded, and a CXR, full blood count, and inflammatory markers were requested. Purulent sputum, if present, was sent for culture and sensitivities. Pneumonia was diagnosed using the British Thoracic Society criteria, with minor modifications (Table 1). As the cough reflex is frequently impaired after stroke, cough was included as part of the symptom complex of lower respiratory tract illness rather than as a mandatory symptom in its own right. Quantification of tachypnoea as a respiratory rate above 25/min and of hypoxia as an oxygen saturation of less than 90% on room air were arbitrarily added to the new focal chest signs criterion to allow consistent scoring (e.g. respiratory rate greater than 25/minute rather than tachypnoea). The white blood cell count (WBC) was added to the systemic features as the BTS clinical symptom complex of aches, pains, and sweating is difficult to ascertain, and easily missed, in stroke patients with a reduced level of consciousness or with dysphasia. To be recognized as a sign of infection, crackles had to be of new onset, coarse, and unilateral or asymmetrically distributed over the lung fields. Fulfilment of all five diagnostic criteria was required for the diagnosis of pneumonia. If a diagnosis of pneumonia was made, all physical signs were recorded on the day of diagnosis. In addition, if pneumonia was diagnosed at night, the vital signs and chest signs at the time of the diagnosis of pneumonia were obtained from the case notes. Clinical signs and interpretation of CXR were corroborated by independent medical staff and radiologists.

**Table 1:** Diagnostic criteria for pneumonia.

Symptoms of an acute lower respiratory tract illness	Cough and /*or*
	At least one other lower respiratory tract symptom *[tachypnoea (respiratory rate > 25 per minute)*, *sputum production or oropharyngeal secretions*, *hypoxia (oxygen saturation ≤ 90% on room air)]*
New focal chest signs on examination	*new inspiratory crackles*
	*bronchial breathing*
	*signs of consolidation*
At least one systemic feature	fever over 38°C
	symptom complex of sweating, rigors, fever and aches and pains
	*leucocytosis (WBC >11000/ml)*
	*leucopoenia (WBC <3000/ml)*
No other obvious cause for the symptoms	
Radiological shadowing which is at least in one segment and not known to be previously present for which there is no other explanation.	

The British Thoracic Society recommendations for definition of community acquired pneumonia^1^ were adapted for this study. Modifications are indicated by italics.

Patients were examined daily during the period of pneumonia. As the study was designed to diagnose pneumonia early rather than to monitor its progression, all clinical signs and symptoms and the inflammatory markers were recorded only on the day of the diagnosis. It was also thought that antibiotic treatment would alter the progression of physical signs and symptoms of pneumonia. Therefore, only the temperature and the inflammatory markers were recorded on the third day of antibiotics (to assess the response to treatment), and at the end of the course of antibiotics. However, presence of purulent sputum was recorded throughout the study, and all samples were sent for cultures. Recurrent pneumonia was diagnosed using BTS guidelines and was defined for this study as pneumonia recurring more than 48 hours after completion of a full course of antibiotic therapy. Reappearance of signs and symptoms of lower respiratory tract infection, increasing inflammatory markers and new or worsening radiological shadowing in CXR were used to diagnose recurrent pneumonia. Other non-respiratory infections were also recorded. A positive urinary culture was required for the diagnosis of urinary tract infection. All available CRP and WBC tests performed during the observation period, irrespective of presence of pneumonia, were recorded. All pneumonias were treated with intravenous antibiotics according to local guidelines and temperatures >38°C were treated with 1g of paracetamol enterally. CRP was measured in a clotted biochemistry sample using a Latex enhanced immuno-turbimetric assay (Siemens Advice 2400), and analysed within 24 hours of collection.

## Statistical Analysis

In the first stage of analysis, CRP was entered in a logistic regression model with pneumonia diagnosis as the outcome variable. The mean of all available CRP values over the first week was used for patients with no pneumonia. The CRP value on the day of, or the closest to the day of, the diagnosis of pneumonia was used in patients with pneumonia. CRP was assessed in terms of its statistical significance as a predictor in the logistic regression model, but also in terms of its predictive strength; this was indicated by the goodness-of-fit of the model, as expressed by the Nagelkerke pseudo-*R*^2^ statistic.

In the second stage of analysis, receiver operating characteristics (ROC) methods were used to identify an appropriate diagnostic threshold for CRP, with an area under the ROC curve (AUC) of one indicating the greatest possible sensitivity and specificity and an AUC of zero denoting the lowest possible sensitivity and specificity. The Youden index (*J*) was also calculated for each observed point on the scale. This statistic expresses, on a 0–1 scale, the maximum difference between sensitivity and 1–specificity (i.e. between the true positive rate and the false positive rate) for a given cut-off score, identifying the diagnostic cut-off that simultaneously maximizes both sensitivity and specificity, providing the maximal diagnostic accuracy.

## Results

Sixty patients were recruited. Baseline characteristics of participants are given in Table 2. The mean age was 77 years, most strokes were ischaemic, the mean (SD) National Institutes for Health Stroke Scale (NIHSS) score was 19.47 (6.14), and 51/60 patients had a total anterior circulation syndrome. None of the participants was intubated or ventilated. The mean (SD) WBC was 9.72 (3.22) cells x 10^9^/l, within normal laboratory range on admission (normal range 4–11 cells x 10^9^/l) but the median (IQR) CRP level was elevated at 9.7 (4.0–15.5) mg/L (normal range 0–5 mg/L). The median time (IQR) from stroke onset to recruitment was 2 (1–3) days.

**Table 2:** Clinical and demographic characteristics of the study sample.

		Pneumonia *n* = 33	No pneumonia *n* = 27	*p* value
Age; mean (SD) (years)		78.12 (10.57)	77.96 (8.54)	.950*
Sex (male); *n* (%)		15 (46)	7 (26)	.118^†^
NIHSS; mean (SD)		20.79 (6.29)	17.37 (5.96)	.036*
CT diagnosis; *n* (%)	Infarction	30 (91)	26 (96)	.405^†^
	Haemorrhage	3 (9)	1 (4)	-
Stroke syndrome; *n* (%)	TACS	29 (88)	22 (82)	.753^†^
	PACS	2 (6)	3 (11)	-
	POCS	2 (6)	2 (7)	-
Comorbidity; *n* (%)	Diabetes	9 (27)	8 (30)	.840^†^
	Hypertension	24 (73)	16 (59)	.271^†^
	Atrial fibrillation	18 (55)	13 (48)	.622^†^
ACE inhibitors; *n* (%)	Yes	12 (36)	6 (22)	.234^†^
Previous lung pathology; *n* (%)	Yes	7 (21)	3 (11)	.488^#^
	COPD	5 (15)	3 (12)	-
	Asthma	2 (6)	0 (0)	-
	Others–fibrosis	0 (0)	0 (0)	-
Baseline chest radiograph; *n* (%)	Abnormal	4 (12)	3 (11)	.999^#^
	COPD	2 (6)	2 (8)	-
	Chronic changes, pulmonary vascular congestion	2 (6)	1 (4)	-
Baseline inflammatory markers	CRP mg/l; median (IQR) ^**^	10.00 (4.65, 13.50)	7.30 (3.80, 24.50)	.821^‡^
	WBC cells x 10^9^/l; mean (SD)	9.99 (3.74)	9.40 (2.47)	.481*

* t test

† chi-square test

‡ Wilcoxon rank sum test

# Fisher’s exact test

** based on n = 33 and n = 27 for the pneumonia and no pneumonia groups respectively

SD: standard deviation, IQR: interquartile range, ACE: angiotensin conversion enzyme, NIHSS–National Institute for Health Stroke Scale, CT: computed tomography, TACS: total anterior circulation syndrome, PACS: partial anterior circulation syndrome, POCS: posterior circulation syndrome, COPD: chronic obstructive pulmonary disease.

There were 44 episodes of pneumonia in 33 patients that fulfilled the diagnostic criteria of this study. The median (IQR) time from stroke onset to the first pneumonia was 3.5 days (3.0–5.0 days). The most commonly encountered symptoms on the day of the diagnosis were tachypnoea and a drop in oxygen saturation to less than 90%. These were observed in 42 (95%) and 41 (93%) episodes of pneumonia respectively (Table 3). Cough and purulent sputum were less commonly encountered in 27 (61%) and 25 episodes (57%) of pneumonia respectively. The mean peak temperature associated with pneumonia was 37.7°C; temperatures >38°C were seen in only 15 episodes (32%). All patients with a temperature >38°C received paracetamol. Of patients who did not have a temperature >38°C, five were given paracetamol during the time of the study, three in the pneumonia group and two in the non-pneumonia group. The commonest physical sign on chest examination was new-onset crackles, observed in 43 episodes (98%) of pneumonia. Signs of consolidation, such as dullness to percussion and bronchial breathing, were seen in only 25 (57%) and 3 (7%) episodes respectively.

**Table 3:** Symptoms, signs and laboratory results on the day of the diagnosis of first and recurrent pneumonias.

	First episode (*n* = 33); *n* (%)	Second episode (*n* = 11); *n* (%)	Total (*n* = 44); *n* (%)
**Signs and symptoms**			
Respiratory rate >25/min	31 (94)	11 (100)	42 (95)
Oxygen saturation <90%	30 (91)	11 (100)	41 (93)
Cough	21 (64)	6 (46)	27 (61)
Purulent sputum	19 (56)	6 (46)	25 (57)
Temperature > 38°C	12 (35)	3 (23)	15 (34)
Chill and rigors	1 (3)	0 (0)	1 (2)
**Chest examination**			
New-onset inspiratory crackles	32 (97)	11 (100)	43 (98)
Signs of consolidation	18 (54)	7 (54)	25 (57)
Bronchial breathing	3 (9)	0 (0)	3 (7)
**Investigations**			
CRP >5 mg/l	33(100)	11 (100)	44 (100)
CRP >10 mg/l	32(97)	11(100)	43 (97)
CRP >30 mg/l	30 (90)	10 (91)	40 (91)
WBC >11 cells x 10^9^/L	29 (85)	9 (69)	38 (86)
WBC < 3 cells x 10^9^/L	1 (3)	0 (0)	1 (2)

CRP: C-reactive protine, WBC: White blood count

One patient who had a temperature < 38°C developed a fever >38°C after commencement of antibiotics. Purulent sputum was observed in a further seven episodes of pneumonia over the next few days after the diagnosis, making a total of 32 (71%) episodes with purulent sputum. In the non-pneumonia group, there were three (11%) patients with isolated tachypnoea and two (7%) patients with hypoxia and tachypnoea in combination. These episodes were due to pre-existing chronic obstructive pulmonary disease (COPD) and episodes of congestive cardiac failure.

Fourteen (30%) episodes of pneumonia had positive sputum cultures, predominantly Gram-negative organisms (n = 10). Other non-respiratory infections were urinary tract infection, (n = 6) and cellulitis (n = 1). An elevated WBC was observed in 38 episodes of pneumonia (86%).The most consistent inflammatory marker on the day of the diagnosis was an increase in CRP from baseline which was seen in 41 episodes of pneumonia (93%). Summary values of CRP are shown in Table 4. Within the logistic regression model, the odds ratio for a one-unit increase in CRP was 1.030 (95% confidence interval [CI] 1.012, 1.048; *p* = 0.001). Thus, an increase of 10 mg/L of CRP increases the likelihood (odds) of pneumonia by 30%. The Nagelkerke *R*^2^ value for the model was 0.395.

**Table 4:** Values of C-reactive protein in the analysis.

	Pneumonia (*n* = 33)	No pneumonia (*n* = 27)
median (IQR)	81.70 (30.50, 131.00)	22.00 (8.85, 30.70)
range	1.60, 240.00	2.70, 155.00

IQR: interquartile range

**Fig 1:**
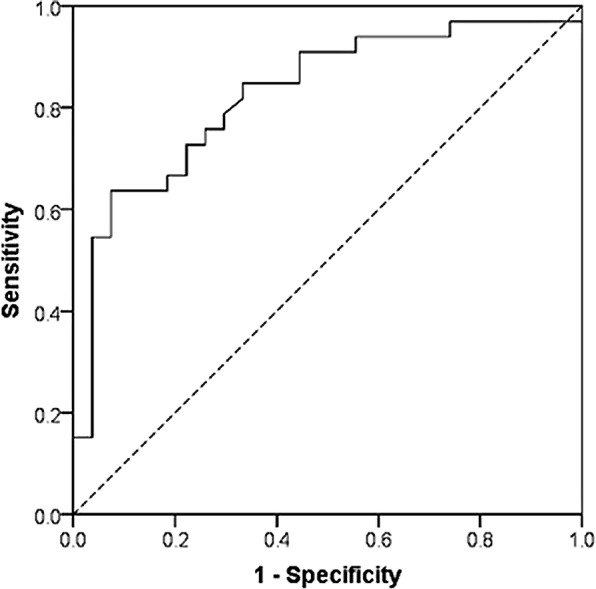
ROC curve for C-reactive protein. Area under the curve = 0.827. A diagonal segment on the curve denotes a tie.

CRP was then taken forward to the second stage of data analysis. From the ROC analysis, the AUC for CRP was 0.827 (95% CI 0.720, 0.933, *p* < .001; Fig 1). Table 5 shows the sensitivity, specificity, positive and negative predictive values, and the Youden index (*J*) for observed values of CRP. The highest combined sensitivity and specificity (*J* of 0.562) was for a CRP value of 64.65 mg/L (sensitivity 0.636; specificity 0.926). However, if high sensitivity (>0.800) is important, the optimal cut-off was 25.60 mg/L (*J* 0.515; sensitivity 0.848; specificity 0.667). Table 6 displays sensitivity, specificity, and positive and negative predictive values for different cut-offs with 95% confidence intervals (calculated by the Wilson method), to indicate the precision of these estimates.

**Table 5:** Sensitivity and specificity of CRP at different cut-off values.

Cutoff	Sensitivity	Specificity	Youden index	PPV	NPV
14.90	.939	.370	.309	.646	.832
17.20	.939	.407	.346	.659	.845
19.70	.939	.444	.383	.674	.856
20.90	.909	.444	.353	.666	.800
21.90	.909	.481	.390	.682	.812
22.45	.909	.519	.428	.698	.824
22.95	.909	.556	.465	.714	.833
23.50	.848	.556	.404	.700	.750
24.60	.848	.630	.478	.737	.772
25.60	.848	.667	.515	.757	.782
27.50	.818	.667	.485	.750	.750
29.50	.788	.704	.492	.765	.731
30.20	.758	.704	.462	.758	.704
30.45	.758	.741	.499	.782	.715
30.75	.727	.741	.468	.774	.690
36.50	.727	.778	.505	.800	.700
45.00	.697	.778	.475	.793	.678
50.00	.667	.778	.445	.786	.657
53.00	.667	.815	.482	.815	.667
55.00	.636	.815	.451	.808	.647
57.25	.636	.852	.488	.840	.657
59.40	.636	.889	.525	.875	.666
64.65	.636	.926	.562	.913	.675
69.50	.606	.926	.532	.909	.658
71.00	.576	.926	.502	.905	.641
74.15	.545	.926	.471	.900	.625
77.65	.545	.963	.508	.947	.634
80.35	.515	.963	.478	.944	.619
84.35	.485	.963	.448	.941	.605
89.50	.455	.963	.418	.938	.591
97.50	.424	.963	.387	.933	.578
105.00	.394	.963	.357	.929	.565
110.50	.364	.963	.327	.923	.553

Only values with both a sensitivity and a specificity of at least .350 are shown. CRP: C-reactive protein, PPV: positive predictive value, NPV: negative predictive value.

**Table 6:** Diagnostic statistics for cutoffs of CRP with 95% confidence intervals (CIs).

Cut-off	Sensitivity (95% CI)	Specificity (95% CI)	PPV (95% CI)	NPV (95% CI)
25.60	0.848 (0.691, 0.933)	0.667 (0.478, 0.814)	0.757 (0.599, 0.866)	0.782 (0.581, 0.903)
64.65	0.636 (0.466, 0.778)	0.926 (0.766, 0.979)	0.913 (0.732, 0.976)	0.675 (0.515, 0.804)

PPV: positive predictive value, NPV: negative predictive value. See the contingency table in supportive information for raw data

## Discussion

The assessment of post-stroke pneumonia in clinical practice can be challenging. Early and accurate diagnosis is necessary for timely and appropriate initiation of antibiotic treatment. The results of this study show that clinical features typically associated with pneumonia in other clinical settings–such as pyrexia >38°C, cough, and purulent sputum–were frequently not seen in early stages of post-stroke pneumonia. This may have been due to an altered level of consciousness, an impaired cough reflex, respiratory muscle paresis and a muted febrile response. An increase in the respiratory rate, a fall in oxygen saturation <90%, and new-onset inspiratory crackles were the most common clinical features of post-stroke pneumonia. However, these signs can also be present in other cardiorespiratory diseases such as atelectasis, pulmonary embolism, and heart failure. There is a potential risk of delay in diagnosis of pneumonia in this patient group, as these common clinical features are considered to be non-specific. This study demonstrates the need for specific guidelines for the diagnosis of post-stroke pneumonia, as some of the key diagnostic features of pneumonia are infrequently seen in patients with pneumonia early after an acute stroke.

Several current guidelines require a temperature >38°C for the diagnosis of pneumonia [1,2]. However, in our study only 15 (34%) episodes of pneumonia were associated with temperatures >38°C. Participants in our study were elderly, and older patients do not always manifest high temperatures with acute infections [6]. Treatment with paracetamol and aspirin could also have muted the temperature response [5], but only 5 patients with a temperature <38°C were given paracetamol during the trial period following stroke. However, while high fevers were uncommon, 36 (86%) of patients with pneumonia in our study had temperatures >37.7°C. Stroke-related brain injury can itself cause a rise in the body temperature up to 37.5°C during the first 12 hours after onset, even in the absence of infection [8]. We would therefore recommend starting investigations for an infection with a temperature >37.7°C, which is higher than the stroke-associated temperature rise, but below levels suggested by guidelines for the diagnosis of pneumonia [1,13].

CRP is an acute-phase protein and has a normal plasma level of <5 mg/L [6]. CRP values above 60–80mg/L within 24 hours of hospitalization are highly sensitive and specific for the diagnosis of a bacterial infection in older people [6, 14]. Raised CRP levels are also seen with non-infective pathologies such as pulmonary embolism, malignancies, trauma, and stroke. Patients in our study had a median (IQR) CRP of 9.70 (4.0, 15.50) mg/L at recruitment. This is comparable to ranges described in an earlier study, which reported CRP values of 4 mg/L on admission and a peak of 18 mg/L at 5–7 days after stroke [8]. CRP and elevated WBC may both be induced by cerebral ischaemia associated with acute stroke in the absence of infection [8]. A recent systematic review of diagnostic approaches to pneumonia complicating stroke in research studies showed that CRP was rarely used in the diagnosis of post-stroke pneumonia, whereas WBC was incorporated more frequently as a diagnostic criterion [4].

In our study 92% of all patients had an elevated CRP (>10 mg/L) within the first week, whether they developed pneumonia or not. This cut-off is therefore not suitable for a diagnosis of pneumonia. The Youden index assumes equal weight for sensitivity and specificity. The highest overall diagnostic accuracy (*J* = 0.562) was observed at a cut-off of 64.65 mg/L, with high specificity (0.926), but relatively low sensitivity (0.636). This would allow for high diagnostic certainty, but miss a significant number of cases. For screening purposes, high sensitivity (above 0.8) is important. The optimal cut-off for this level of sensitivity was 25.60 mg/L (*J* 0.515; sensitivity 0.848; specificity 0.667). This is well above the level expected in patients with acute stroke [8], and could be used to prompt investigations for pneumonia. This level could also be used to initiate treatment, if combined with clinical signs such as hypoxia, tachypnoea, and new onset of crepitations. This strategy will reduce unnecessary administration of antibiotics for non-infectious conditions and prevent complications induced by antibiotics, including emergence of antibiotic resistance. In addition, our study shows that for each 10 mg/L increase in CRP, the likelihood (odds) of pneumonia increases by 30%. While a cut-off of 25.60mg/L is the optimal value for screening, our findings also show that a CRP value of 64.65 mg/L or above is sufficiently specific to start treatment irrespective of clinical findings while awaiting formal confirmation of post-stroke pneumonia.

As far as we know this is the first study that has reviewed the early symptomatology of post-stroke pneumonia, investigated the role of CRP as a potential biomarker for early diagnosis (rather than for stroke-associated infections in general), and established a cut-off value for CRP. Strengths of this study are that it was conducted prospectively and that the daily physical examination was done by the same team in each case. This led to high consistency and also high diagnostic yield. The cut-off values identified in this study have the potential to inform clinical decision–making in stroke patients with non-specific clinical signs and symptoms, but will need to be confirmed in an independent study.

Our study has limitations. The number of participants was small, and confidence intervals around the estimates of sensitivity, specificity, positive and negative predictive values are therefore somewhat wide. All patients were enrolled from a single centre and local factors influencing pneumonia incidence cannot be excluded. The incidence of pneumonia in our cohort is high. This is likely to be due to selection of a high-risk group and the rigorous and consistent approach to clinical diagnosis. In addition, clinical findings were confirmed in all cases by new infiltrates on the chest x-ray. However, radiographic opacification can be seen in several other non-infective pathologies such as atelectasis and pulmonary embolism. As the chest radiograph was only one of five diagnostic criteria, over-diagnosis of pneumonia due to non-specific infiltrates is less likely. It may be argued that the modifications made to the BTS diagnostic criteria for pneumonia, especially the change of ‘cough *and* other lower respiratory tract symptoms’ to ‘cough *or* other lower respiratory tract symptoms’ could have been a reason for the high incidence of pneumonia. However, as severe stroke is associated with an impaired cough reflex, the inclusion of cough as a mandatory symptom would have led to under-diagnosis in this most vulnerable patient population. This modification is in line with stroke-specific criteria for the diagnosis of pneumonia [4, 13], which include cough as part of a complex of respiratory symptoms rather than as an essential criterion in its own right. This modification is balanced by more robust, detailed requirements in other diagnostic criteria (clear definitions of tachypnoea, oxygen saturation, chest signs), which are likely to mitigate the risk of over-diagnosis. Dividing patients into those with and without pneumonia using fixed criteria is necessary for initiation of treatment and diagnostic classification, but may not reflect real-life pathology. Pneumonia develops gradually, and some signs and symptoms are likely to appear earlier than others. Levels of inflammatory makers increase over time, and cut off-values are to some degree arbitrary. However, Table 5 shows how diagnostic specificity rises with the level of CRP, increasing diagnostic confidence. The results of our study have not been validated in an independent cohort of stroke patients. The findings need to be confirmed in a larger, multicentre study with external validation in mixed populations with a wider range of stroke severities.

## Conclusion

Patients with severe stroke frequently do not manifest key diagnostic features of pneumonia, such as pyrexia, cough and purulent sputum, early in their illness. As these feature in most diagnostic algorithms for pneumonia, there is a potential risk of under-diagnosis in this patient group. Our results suggest that a CRP value >25 mg/L should prompt investigations for pneumonia while values above 65 mg/L have the highest diagnostic accuracy to justify consideration of this threshold as a diagnostic marker of post-stroke pneumonia. These findings need to be confirmed in larger, multicentre study with external validation in mixed populations with a wider range of stroke severities.

## Supporting Information

S1 Appendix Supplemental data. (PDF)

S1 Table Contingency Table. (PDF)
