# Supplementary material for: Early Diagnosis of Pneumonia in Severe Stroke: Clinical Features and the Diagnostic Role of C-Reactive Protein
Source: PLoS One. 2016 Mar 3;11(3):e0150269. doi: 10.1371/journal.pone.0150269 (PMC4777448; doi:10.1371/journal.pone.0150269)
Supplement: S1 Appendix — (PDF) [file pone.0150269.s001.pdf]

Supplemental Data

Cough			D1	D2	D3	D4	D5	D6	D7	D8	D9	D10	D11	D12	D13	D14	D15	D16	D17	D18	D19	D20	D21
Rand No	pneumonia																						
1	YES	N		Y	Y										Y			Y					
2	YES	N			N		Y								N								
3	YES	N			N		Y					N	N										
4	NO	N			N			N															
5	NO	N			N			N															
6	YES	N			N				Y	Y					N								
7	NO	N			N			N						N									
8	NO	N			N			N							N								
9	NO	N			N			N															
10	NO	N			N			N															
11	YES	N		Y	Y																		
12	YES	N	N		N										N								
13	YES	N	N		N									N									
14	NO	N			N			N															
15	NO	N			N			N							N								
16	NO	N			N			N							N								
17	YES	N		N	N							N	N		N								
18	YES	N	N		N	N								N	N								
19	NO	N			N			N															
20	YES	N		N	N			N															
21	NO	N			N			N															
22	NO	N			N			N															
23	YES	N	Y		Y								Y										
24	YES	N		N	N		N																
25	YES	N	N		N	N								N	N							N	
26	YES	N		N	N							N	N										
27	YES	N	N		N			N							N								
28	NO	N			N																		
29	YES	N			N		Y								Y				Y	Y			
30	YES	N		Y	Y																		
31	yes	N			Y																		
32	yes	N			N																		
33	yes	N	Y	Y	Y			N							N						N		
34	yes	N		Y	Y						Y												
35	no	N			N	N		N															
36	yes	N		N	N			Y															
37	no	N			N			N															
38	NO	N			N			N							N							N	
39	no	N			N			N															
40	no	N			N			N															
41	no	N			N			N															
42	no	N			N			N							N								
43	yes	N		Y	Y										N								
44	no	N	N		N			N															
45	yes	N		Y	Y										N					Y			
46	yes	N			Y	Y									N								
47	yes	N		Y	Y										N								
48	yes	N	Y	Y	Y																		
49	no	N			N			N															
50	yes	N	Y	Y	Y										N								
51	yes	N		Y	Y										N		N						
52	no	N				N		N							N								
53	no	N			N			N							N								
54	yes	N	Y	Y	Y										N				N				N
55	no	N			N			N															
56	no	N			N			N															
57	yes	N			Y										N								
58	yes	N			N			N															
59	yes	N	Y	Y	Y			Y							N				Y				
60	yes	N		Y	Y			N															

**Purulent**	**Sputum**		D1	D2	D3	D4	D5	D6	D7	D8	D9	D10	D11	D12	D13	D14	D15	D16	D17	D18	D19	D20	D21
Rand No	pneumonia																						
1	YES			Y	Y			Y										Y					
2	YES						N	N															
3	YES						N	N				N	N										
4	NO							N															
5	NO							N	N														
6	YES									Y													
7	NO							N															
8	NO							N															
9	NO							N															
10	NO							N															
11	YES			Y	Y																		
12	YES		N		N																		
13	YES		N		N																		
14	NO							N															
15	NO							N															
16	NO							N															
17	YES			Y								N	N										
18	YES		N			N		N						N									
19	NO							N															
20	YES			N																			
21	NO							N															
22	NO							N															
23	YES		N										N										
24	YES						Y																
25	YES					Y								Y									
26	YES			N	N							N	N										
27	YES		N																				
28	NO				N																		
29	YES		N				N	N											N	N			
30	YES			Y				N															
31	yes				Y			N															
32	yes				N																		
33	yes			N				N													Y		
34	yes			N				N			Y												
35	no					N		N															
36	yes				Y			Y															
37	no							N															
38	NO							N															
39	no							N															
40	no							N															
41	no							N															
42	no							N															
43	yes			Y				N															
44	no																						
45	yes			Y	Y			Y												Y			
46	yes				Y	Y		Y															
47	yes			N				N															
48	yes		Y		Y			Y															
49	no							N															
50	yes			N	N			N															
51	yes				Y			Y									N						
52	no					N		N															
53	no				N			N															
54	yes		N	N				N											N				
55	no							N															
56	no							N															
57	yes				Y			Y															
58	yes				Y			Y															
59	yes		Y	Y				N											Y				
60	yes			Y				Y															

Signs	of Consolidations		D1	D2	D3	D4	D5	D6	D7	D8	D9	D10	D11	D12	D13	D14	D15	D16	D17	D18	D19	D20	D21
Rand No	pneumonia																						
1	YES				Y													Y					Y
2	YES						Y																
3	YES						N						N										
4	NO							N															
5	NO							N															
6	YES									Y													
7	NO							N															
8	NO							N															
9	NO							N															
10	NO							N															
11	YES				Y																		
12	YES		N		N																		
13	YES		N		N																		
14	NO							N															
15	NO							N															
16	NO							N															
17	YES			Y								N											
18	YES					Y								Y									Y
19	NO							N															
20	YES			Y																			
21	NO							N															
22	NO							N															
23	YES		Y										N										N
24	YES			N			N																
25	YES					Y								Y									
26	YES				Y								Y										
27	YES		Y																				
28	NO							N															
29	YES						Y													Y			
30	YES			Y																			
31	yes				N																		
32	yes				N																		
33	yes			N																	N		
34	yes			Y							Y												Y
35	no					N																	
36	yes				N			N															
37	no							N															
38	NO							N															
39	no							N															
40	no							N															
41	no							N															
42	no							N															
43	yes			Y																			
44	no							N															
45	yes			Y	Y															Y			
46	yes			N		N																	
47	yes			N																			
48	yes		N		N			N															
49	no							N															
50	yes		N		N																		
51	yes				Y												N						
52	no					N		N															
53	no				N																		
54	yes		Y	Y															N				N
55	no							N															
56	no							N															
57	yes	N			N																		
58	yes	N			N																		
59	yes		N	N												N			N				
60	yes	N		N																			

Inspiratory	crackles		D1	D2	D3	D4	D5	D6	D7	D8	D9	D10	D11	D12	D13	D14	D15	D16	D17	D18	D19	D20	D21
Rand No	pneumonia																						
1	YES	N		Y	Y		Y	Y							N		Y	Y	Y	Y	Y	Y	
2	YES	N			N	Y	Y	Y							N								
3	YES	Y			N	Y	Y	Y	Y			Y	Y	Y	Y								
4	NO	N			N		N	N															
5	NO	N			N		N	N															
6	YES	N			N			N	Y	Y	Y	Y	Y	Y	Y			N				N	
7	NO	N			N		N	N						N									
8	NO	N			N		N	N						N	N				N				
9	NO	N			N		N	N															
10	NO	N			N		N	N				N	N										
11	YES	N		Y	Y		Y	Y	Y														
12	YES	N	Y	Y	Y	Y	Y	Y						N	N						N	N	
13	YES	Y	Y	Y	Y	Y	Y	Y						N	N								
14	NO	N			N		N	N															
15	NO	N			N		N	N															
16	NO	N			N		N	N						N	N						N	N	
17	YES	N	Y	Y	Y	Y	Y	Y			N	Y	Y	Y	Y								
18	YES	N	Y	Y	Y	Y	Y	Y					N	N	Y	Y					Y	Y	
19	NO	N			N		N	N	N					N	N						N	N	
20	YES	Y	Y	Y	Y	Y	Y	Y															
21	NO	N			N		N	N			N												
22	NO	Y			N		Y	Y		N													
23	YES	N	Y	Y	Y	Y	Y					Y	Y										
24	YES	N		Y	N	Y	Y																
25	YES	N	N	N	Y	Y	Y	Y						Y	Y						Y	Y	
26	YES	N		Y	Y	Y	Y	Y				Y	Y								Y	Y	
27	YES	N	Y	Y	Y	Y	Y	Y						N	N								
28	NO	N		N	N																		
29	YES	N	Y	Y	N	Y	Y			Y				Y	Y			N	Y	Y	Y	Y	
30	YES	N		Y	Y	Y	Y	Y															
31	yes	N		Y	Y	Y	Y	Y						N	N						N	N	
32	yes	N		Y	Y	Y	Y																
33	yes	N	Y	Y	Y	Y	Y	Y						Y	Y					N	Y	Y	
34	yes	N	Y	Y	Y					N	Y	Y											
35	no	N			N	N																	
36	yes	N			Y	Y		Y				N											
37	no	N			N		N	N				N											
38	NO	N			N		N	N						N	N						N		
39	no	N			N		N	N															
40	no	N		N	N		N	N			N												
41	no	N			N		N	N			N												
42	no	N			N		N	N						N	N						N	N	
43	yes	N	Y	Y	Y	Y	Y	Y			Y			Y	Y								
44	no	N			N		N	N															
45	yes	N		Y	Y	Y	Y	Y							N				Y	Y	Y	Y	
46	yes	N		Y	Y	Y	Y	Y							N								
47	yes	N		Y	Y	Y	Y	Y						Y	Y						N	N	
48	yes	N	Y	Y	Y	Y	Y	Y															
49	no	N			N		N	N															
50	yes	N	N		Y	Y	Y	Y						Y	Y								
51	yes	Y	Y	Y	Y	Y	Y	Y							N	N	N				N	N	
52	no	N			N	N	N	N						N	N						N	N	
53	no	N		N	N	N	N	N															
54	yes	N	Y	Y	Y	Y	Y	Y							N			Y	Y	Y			
55	no	N			N		N	N															
56	no	N			N		N	N															
57	yes	N		Y	Y	Y	Y	Y						Y	Y								
58	yes	N		Y	Y	Y	Y	Y			Y												
59	yes	N	Y	Y	Y	Y	Y	Y							Y			N	Y	Y	Y	Y	
60	yes	N		Y	Y	Y	Y	Y															

Temperature			D1	D2	D3	D4	D5	D6	D7	D8	D9	D10	D11	D12	D13	D14	D15	D16	D17	D18	D19	D20	D21
Rand No	pneumonia	Temp																					
1	YES	36.0			37.9			37.9							36.9		36.7	37.9				37.8	
2	YES	-			36.1		38.0	36.0							36.5								
3	YES	35.0			36.8		37.9	37.2				36.8	37.5		36.0							35.0	
4	NO	36.6			37.0			37.2															
5	NO	37.0			36.9			37.0															
6	YES	36.7			36.9			37.2	37.2	39.0					37.0							37.5	
7	NO	36.8			37.0			37.0							36.8								
8	NO	36.0			36.9			37.0															
9	NO	36.8			37.1			37.0															
10	NO	36.5			36.8			36.8							36.8								
11	YES	36.8			38.9			37.5															
12	YES	36.0			37.5			37.0							36.9							36.9	
13	YES	36.8			37.8			37.0															
14	NO	36.8			36.9			37.0															
15	NO	36.8			37.1			37.1															
16	NO	37.1			37.0			37.1							37.5							36.0	
17	YES	36.5		37.7	37.6			37.4			37.0	37.8			37.1								
18	YES	36.5			37.1	37.6		37.3					36.9	37.9	37.5							36.5	
19	NO	36.7			36.0			36.5							36.8							37.2	
20	YES	37.0		37.8				-															
21	NO	36.8			36.9			36.8															
22	NO	36.8			37.0			37.0															
23	YES	36.9	39.0		37.5			37.0				37.5	37.9		36.0								
24	YES	37.1			36.9		37.6	37.1															
25	YES	37.0			36.9	38.5		38.5					36.7	38.0	38.0							38.0	
26	YES	-			37.8			37.0				36.5	37.5		37.0							36.0	
27	YES	36.9	37.8		37.2			37.2							37.0								
28	NO	37.0			36.9			37.0															
29	YES	36.9			37.0		37.8	37.4							36.9				36.7	37.6		37.2	
30	YES	36.0		38.9	38.7			38.9															
31	yes	37.0		36.9	37.7			37.0							36.5							36.5	
	yes	36.0		37.0	39.0			37.0															
33	yes	36.5		37.8	37.5			37.0							36.4					36.0	37.9	37.0	
34	yes	36.7		37.8	37.5			37.3		36.0	37.7				37.0								
35	no	36.0			37.0	37.8		37.5															
36	yes	36.0			37.9			37.0							36.5								
37	no	37.0			37.0			37.5							37.0								
38	NO	36.0			36.9			-							-							-	
39	no	36.6			37.0			37.0															
40	no	36.5			37.0			37.0															
41	no	36.7			36.9			37.0							37.0								
42	no	36.5			36.7			36.5							36.0							36.5	
43	yes	37.0		38.0	38.0			38.0							37.5								
44	no	37.0			36.8			37.0															
45	yes	36.7			38.0			38.0							37.0				36.9	37.9		37.8	
46	yes	36.8			37.0	37.8		37.3							37.0								
47	yes	36.4			37.5			37.5							36.5							36.5	
48	yes	36.8			37.8			37.2															
49	no	36.5			36.7			37.0															
50	yes	36.0			37.9			37.6							37.0								
51	yes	36.0			37.8			37.5							37.5	37.5	37.9					36.5	
52	no	36.7			37.3	37.9		36.8							36.8								
53	no	36.5			37.8			36.5							36.5								
54	yes	37.0		38.5				37.5							37.5			36,5	37.9			37.5	
55	no	36.0			37.1			37.5															
56	no	36.5			37.0			37.0															
57	yes	36.8			37.9			37.9							37.0								
58	yes	36.5			38.0			38.0															
59	yes	36.0		38.0	38.0			38.0							37.5			36.5	37.5			37.5	
60	yes	37.0		38.9	38.0			38.0															

Respiratory	rate		D1	D2	D3	D4	D5	D6	D7	D8	D9	D10	D11	D12	D13	D14	D15	D16	D17	D18	D19	D20	D21
Rand No	pneumonia	RR																					
1	YES	18		25	35			28							20		22	28				22	
2	YES	18			18		30	20							18								
3	YES	16			20	25	28	18				24	28										
4	NO	20			18			21															
5	NO	14			16			14															
6	YES	17			17			20	25	32					24							19	
7	NO	16			16			15															
8	NO	18			16			16															
9	NO	17			17			17															
10	NO	18			16			18							16								
11	YES	17		25	30																		
12	YES	16		25	34			16							15							16	
13	YES	18		24	32			20															
14	NO	18			17			17															
15	NO	18			16			16															
16	NO	18			16			18							18							18	
17	YES	19		32	32			19			24	30			18								
18	YES	18			25	34		19					24	28	19							18	
19	NO	18			16			15							16							17	
20	YES	17		38	30																		
21	NO	16			16			16															
22	NO	18			16			16															
23	YES	24	36		26			24				24	38		28								
24	YES	19			20	25	28	19															
25	YES	18			22	28		25					22	28	20							25	
26	YES	16			32			24				22	28		22							18	
27	YES	18	30		28			18							16								
28	NO	18			20			16															
29	YES	19			20		32	25							20				24	28		20	
30	YES	19	24	32	30			19															
31	yes	18		24	32			18							17							15	
32	yes	18		26	38			28															
33	yes	17	20	28	28			18							16					24	30	24	
34	yes	18	18	28				21		25	32				24								
35	no	18			18	20		20															
36	yes	18		18	24			16							17								
37	no	18			16			17							17								
38	NO	16			16			16							16							16	
39	no	17			16			17															
40	no	19			18			18															
41	no	16			18			15							15								
42	no	18			18			15							15							16	
43	yes	18	20	28	28			17							16								
44	no	16			18			16															
45	yes	18		20	28			22							20				18	28		22	
46	yes	16			18	28		16							16								
47	yes	19			16			22							16							16	
48	yes	18		22	30			17															
49	no	17			18			17															
50	yes	19		28	28			18							18								
51	yes	18		18	28			20							16		20					18	
52	no	22			16	18		18							17								
53	no	16			18			16							16								
54	yes	17	18	28	28			18							18			24	28			16	
55	no	18			18			16															
56	no	18			18			16															
57	yes	16		18	28			18							16								
58	yes	18		18	30			20															
59	yes	18	20	32	30			18							16			22	28			18	
60	yes	18	18	36	30			16															

WBC			D1	D2	D3	D4	D5	D6	D7	D8	D9	D10	D11	D12	D13	D14	D15	D16	D17	D18	D19	D20	D21
Rand No	pneumonia	WBC																					
1	YES	10.5						27		20			12		15.3							16.2	
2	YES	8						2.3		6.3			6		-4.1								
3	YES	11.9		10.9				13.5							29.2	27						-	
4	NO	11			11			11.1															
5	NO	8.1				8.4		10.2															
6	YES	11.2						14.1		23				19									
7	NO	8.5						11.1															
8	NO	9.9						11						11.2				9					
9	NO	5.4						4.6			7.7		8.5										
10	NO	6.8						7.4							6.5								
11	YES	9.4			13.1			14.1															
12	YES	10.7						10							7.7			7.1				6.3	
13	YES	8.9			14.1	12.4		12.8															
14	NO	9						9.8															
15	NO	7		11	7	9		9.1															
16	NO	8.3			11			7.8		12		11			11			11				10.9	
17	YES	4						11.6							6.2								
18	YES	7				12							11									9.6	
19	NO	9.5						10.4							9.3							8.1	
20	YES	11.4	13	20.4	17	13																	
21	NO	10.6			9.7			10.1		10.3													
22	NO	5.9			5.7			5.5															
23	YES	9.1	14	18	15	12				10.6			15										
24	YES	11.5				13																	
25	YES	9.7				12.8		13.4					13	14			13	12	12	10.5		14.1	
26	YES	11			7.7	10.9		11.3		7.7	10.5		17									9.2	
27	YES	7.8		11.9	12				8						10.5								
28	NO	10.7					12																
29	YES	11.1				15	14	15.1	11.7		11.6			11.6					13	13		11.2	
30	YES	16		22		14																	
31	yes	13	12.4			12									10.5							6.7	
32	yes	12			16	15.3		15.9															
33	yes	8.7		8.8	7	5	7	8.3							9.3						18.2		
34	yes	9.3	11.7		9.6			11.7	9	7	6	5			5.5								
35	no	9.6		9		22	20	20.1															
36	yes	7.3	10.4		15.3		9.4	11.9		8.1					8.1								
37	no	11.4			8.5		9.6	11.4				8.5											
38	NO	8.2						9.6							10.5							9.3	
39	no	10.4						9.3															
40	no	8.2						8.2															
41	no	14.9						10.9															
42	no	8.8						8							6.3							7.9	
43	yes	13					18	16.9	14					16	18								
44	no	9.2				9.8		10.3															
45	yes	10.1			15		13.6		11.1		12.4				20.9				9.7		14		
46	yes	7.6			13	15			11						13.8								
47	yes	6.5			6.4			6.5			7.1		4.4		6.4			5.1		4.1		4.4	
48	yes	10.7			12			11															
49	no	8.4				9.9																	
50	yes	7.3			11			9.5			10.8		11		9.3								
51	yes	2.8		2.6		3	3.2	3.3			4.1				2.9				1.5	1.8	1.9	3.1	
52	no	17			15			13.6			13				11.3								
53	no	10.5			8.9			5.5			8.9		8		8								
54	yes	9		15	22	18	16						16		17.2				14			10.3	
55	no	9.4			11.9			9.6															
56	no	10.5						7															
57	yes	10.8			22		15	13	14					10.9									
58	yes	7.6		22	22	23		20.3															
59	yes	25			41		35		19						35					23		19.5	
60	yes	6.3			10		5																

CRP		D1	D2	D3	D4	D5	D6	D7	D8	D9	D10	D11	D12	D13	D14	D15	D16	D17	D18	D19	D20	D21
Rand No	pneumonia	CRP																				
1	YES	-					203	307		313					146		163					273
2	YES	3.9						9							3.5							
3	YES	10						23			36			98	94							30
4	NO	-						56														
5	NO	7.6						12														
6	YES	-						124		183				305								
7	NO	57						60														
8	NO	4.5						6.3										12				
9	NO	10				6																
10	NO	-						31				22										
11	YES	-		109	141			207														
12	YES	2.1			103			10.3							22							13
13	YES	11			23			7		8.3	5.6											
14	NO	4.4						46														
15	NO	1.6			13			2.1														
16	NO	2.6				37		7.7			12				12.2		6.9					14
17	YES	1.6						10.6							10.6							
18	YES	7.1				87							162									9.7
19	NO	2						42							7.9							7.3
20	YES	-		30.5																		
21	NO	10.2						28		6.8												
22	NO	3.6			1			3.6														
23	YES	-	79	164	138		48		44			62	165									
24	YES	3.7				29		20.7														
25	YES	9.7				240	239		162					154				83	51		12	
26	YES	34			69								79		23							8.1
27	YES	26													14							
28	NO	1.3		10																		
29	YES	3			91			70			36				45		21		30			24
30	YES	25		54				54														
31	yes	5.5	14		107										8							20
32	yes	-		48	72																	
33	yes	12		131	129				39				22		22			76			290	
34	yes	5.2	71	235							216											
35	no	10			64	155																
36	yes	13			114		102	61		32												
37	no	37				43		28				20										
38	NO	15				33									-							-
39	no	6.1						1.7														
40	no	29						19														
41	no	36						7.5														
42	no	-						17.8			21				13.9							2.9
43	yes	10.3		48				53						60	58							
44	no	1.7						59														
45	yes	13			208		124		84			45			22		12			97		94
46	yes	4			117	92			68		40				40.2							
47	yes	4				52									-							31
48	yes	10			30			4														
49	no	4				4		-														
50	yes	4.8			130		160				72				22.6							7
51	yes	17		170	180	258	105	53		53					29			77	258	193	138	78
52	no	35		51				95							51							
53	no	7		13			8								8							
54	yes	4.5		20				17							43				54	56		38
55	no	21					37															
56	no	28			9.8			31														
57	yes	24			81.7	81.3	63		51	40		24			15.6							
58	yes	14			135																	
59	yes	16		128									4		10							24
60	yes	5.5		42			4															

Oxygen	saturations		At Rand	D2	D3	D4	D5	D6	D7	D8	D9	D10	D11	D12	D13	D14	D15	D16	D17	D18	D19	D20	D21
Rand No	pneumonia	O2 sat																					
1	YES	96		90	84	89	90	84	90					90	85		90	84	84			84	
2	YES	96			93	94	90	90	91	91					95								
3	YES	98			95	96	78	89	90	92		91	82	89	82								
4	NO	97			97			98															
5	NO	96			96			97															
6	YES	94			92		92	92	92	89	90	90			89							93	
7	NO	97			96			97							97								
8	NO	96			95	95		-															
9	NO	95			93			95															
10	NO	93			93			96							100								
11	YES	98		94	68	85	87	85															
12	YES	96		93	88	90	91	95							97							97	
13	YES	96		93	88	90	90	94															
14	NO	94			93			94															
15	NO	96	95		94			97															
16	NO	92	93		94			98							96							98	
17	YES	98	95	86	90			93			93	88	90		93								
18	YES	92			91	86		87					92	88	88							94	
19	NO	95	93		93			95							94							96	
20	YES	99	96	88	90			-															
21	NO	98	97		96			97															
22	NO	88			96			97															
23	YES	93	80	89	90			88				92	87		84								
24	YES	96			94	92	88	96															
25	YES	97			93	80		84					92	88	94							84	
26	YES	98		91	80			93				93	88		93							97	
27	YES	99	88	90	90			98							98								
28	NO	98	96		96			96															
29	YES	95			93	91	85	90							95				94	89		91	
30	YES	97	90	85	89	89		93															
31	yes	96		92	86	89		90							96							96	
32	yes	97		91	80	89		84															
33	yes	96	90	88	89			89							97					93	86	88	
34	yes	94	90	88	89			88	90	91	88	88			88								
35	no	97			95	95		90															
36	yes	96		96	93	92		95							95								
37	no	95	94		93			97							97								
38	NO	95			93			95							95							95	
39	no	97	95		94			98															
40	no	96	95		93			95															
41	no	95	94		94			96							95								
42	no	96	94		93			94							94							97	
43	yes	98	91	87	90		92	95							95								
44	no	95	95		94			98															
45	yes	96		91	83	89		93							95				90	86	90	91	
46	yes	97			90	90	90	92							96								
47	yes	97					88	90							98							95	
48	yes	96		93	90	90		97															
49	no	96	95		95			97															
50	yes	97		91	88	90		96							96								
51	yes	95		91	89	90		95							95		94	94				??	
52	no	94			95	94		96							97								
53	no	97		95	95			96							96								
54	yes	98	90	85	90			94							97			93	88	90		99	
55	no	96	94		94			96															
56	no	95	95		93			96															
57	yes	96		96	93	93		93							95								
58	yes	95		95	89	90		95															
59	yes	97	90	82	89			88							94			94	88			??	
60	yes	94	94	90	90			94															

Keys

	Onset of pneumonia
	onset of other infections

2nd pneumonia episode in patient no 17 and 18 and the 1st pneumonia episode in patient no 47 were excluded in analysis as detailed in manuscript.
