# Supplementary material for: Early Diagnosis of Pneumonia in Severe Stroke: Clinical Features and the Diagnostic Role of C-Reactive Protein
Source: PLoS One. 2016 Mar 3;11(3):e0150269. doi: 10.1371/journal.pone.0150269 (PMC4777448; doi:10.1371/journal.pone.0150269)
Supplement: S1 Table — (PDF) [file pone.0150269.s002.pdf]

S1 Table. Contingency Data

Test result		Diagnosis	
		positive	negative
For CRP ≥ 25.60	positive	28	9
	negative	5	18
For CRP ≥ 64.65	positive	21	2
	negative	12	25

CRP – C-reactive protein
